# Can use of pictograms reduce liquid medication administration errors by mothers? An interventional study

**DOI:** 10.1186/s40359-021-00584-9

**Published:** 2021-06-25

**Authors:** Pawan Patidar, Aditya Mathur, Ashish Pathak

**Affiliations:** 1 Department of Paediatrics, Ruxmaniben Deepchand Gardi Medical College, Ujjain, Madhya Pradesh 456010 India; 2 Department of Women and Children’s Health, International Maternal and Child Health Unit, Uppsala University, Uppsala, Sweden; 3 Department of Global Public Health, Health Systems and Policy: Medicines Focusing Antibiotics, Karolinska Institutet, Stockholm, Sweden

**Keywords:** Dose administration, Dosing errors, Dosing devices, Pictograms

## Abstract

**Background:**

Liquid medication dosing errors (LMDE) made by caregivers affect treatment in children, but this is not a well-studied topic in many low-and middle-income countries including in India.

**Methods:**

An intervention study was conducted among mothers attending a pediatric outpatient clinic of a tertiary care setting in Ujjain, India. The mothers randomly measured 12 volumes of a paracetamol liquid preparation by using a dropper (0.5 and 1 mL), measuring cup (2.5 and 5 mL), and calibrated spoon (2.5 and 5 mL) each with two instructions—oral-only measurement session (OMS) and oral plus pictogram measurement session (OPMS, the intervention). The main outcome was dosing error prevalence. The effectiveness of the intervention was assessed by measuring effect size. Risk factors for maximum LMDE were explored using backward multivariate logistic regression models. A *P* value of < 0.05 was considered statistically significant.

**Results:**

In total, 310 mothers [mean (± SD) age, 30.2 (± 4.18) years] were included. LMDE prevalence in the OMS versus OPMS for dropper 0.5 mL was 60% versus 48%; for l mL dropper was 63% versus 54%; for 2.5 mL cup 62% versus 54%; for 2.5 calibrated spoon 66% versus 59%; 5 mL cup 69% versus 57%; and 5 mL calibrated spoon 68% versus 55%. Comparing OMS with OPMS, underdosing was minimum with the calibrated spoon for 2.5 mL (OR 4.39) and maximum with the dropper for 1 mL (OR 9.40), and overdosing was minimum with the dropper for 0.5 mL (OR 7.12) and maximum with the calibrated spoon for 2.5 mL (OR 13.24). The effect size (d_Cohen_) of the intervention OPMS was 1.86–6.4. Risk factors for the most prevalent dosing error, that is, with the calibrated spoon for 2.5 mL, were increasing age of the mother (aOR 1.08; *P* = 0.026) and nuclear family (aOR 2.83; *P* = 0.002). The risk of dosing errors decreased with higher education of the mothers.

**Conclusions:**

Pictograms can effectively minimize LMDE even in less educated mothers.

**Supplementary Information:**

The online version contains supplementary material available at 10.1186/s40359-021-00584-9.

## Background

The American National Coordinating Council for Medication Error Reporting and Prevention defines medication errors as “any preventable event that may cause or lead to inappropriate medication use or patient harm while the medication is in the control of the healthcare professional, patient, or consumer. Such events may be related to professional practice, healthcare products, procedures, and systems including prescribing; order communication; product labelling, packaging and nomenclature; compounding; dispensing; distribution; administration; education; monitoring; and use” [1].

Oral liquid medications are often preferred for children because of their ease of administration. Weight-based dosing is a norm in pediatric prescribing, and liquid medications provide a pediatrician more flexibility and ease in prescribing individualized dosing to children having different body weights. This individualized and accurate dosing decides treatment efficacy and effectiveness [2]. Studies have suggested that over 40% caregivers make some liquid medication dosing errors (LMDE) both in resource-rich and resource-poor settings [2–10]. Medication errors may contribute toward treatment failure and many other adverse events including death [11].

LMDE occur mostly due to lack of experience among parents in administering liquid medications or due to the dosing device [4, 12]. Liquid preparations are more vulnerable for dosing errors as they are more susceptible to dosage errors associated with the dosing device [9]. Many caregivers, including those in low- and middle-income countries (LMICs), commonly use substandard household items such as teaspoons and tablespoons or cups for measuring and administering liquid medications instead of standard liquid medication delivery devices such as medicine cups, droppers, calibrated spoons, and syringes [6, 9, 13]. These standard delivery devices may be lost by parents at home or become dirty, especially in LMICs. An Indian study showed that a kitchen spoon was used commonly by caregivers for measuring liquid medications, resulting in 40% parents making dosing errors in pediatric liquid formulations [6].

Studies conducted in high-income countries have reported that among the various strategies used for reducing medication errors, the pictogram-based intervention is found to be the most beneficial and is used for medication counseling, which resulted in reduced medication dosing errors and improved medication and dosing adherence among caregivers with a low socioeconomic status as well as with lower education [4, 14–17]. However, evidence that pictograms will be an effective intervention in LMICs, including India, is lacking. Therefore, the research question addressed in the present study was: what is the prevalence of liquid medication errors with various measuring devices when oral only instructions were used to instruct mother’s about liquid medication dosing versus pictograms along with oral instructions, under study conditions in Ujjain, India? and can pictograms effectively minimize dosing errors?

## Methods

### Study site and settings

This cross-sectional study was conducted during March–May, 2017 in the Ujjain district of the state of Madhya Pradesh (MP), India. MP is the fifth largest Indian state and has a low human development index of 0.624 [18]. Ujjain has a population of 1.9 million, with approximately 61% people living in rural areas [19]. Other demographic characteristics of the district are provided in National Family Health Survey-4 [19]. The study was conducted between 9:00 a.m. to 1:00 p.m. in the pediatric outpatient department (OPD) of RD Gardi Medical College, Ujjain, a tertiary care institute, managed by a not-for-profit trust. Data were collected from the OPD for at least two days per week to cover a full week. Thus, a convenience sampling of mothers presenting with their children to the pediatric OPD was performed. The mothers were approached in the outpatient waiting area and were asked to participate in the study after describing the study. Written informed consent in Hindi was obtained from the participating mothers.

### Inclusion and exclusion criteria

Mothers of children presenting for care in the pediatric clinic and those who were responsible for medication administration in the household were included. Mothers with a child presenting with an emergency, those with auditory–ocular problems, and those refusing to participate were excluded.

### Sample size calculation

The sample size was calculated using Stata (Version 12.0, Statacorp. Texas, USA). We assumed that parents receiving the text plus pictogram instruction would have 20% less LMDE than those receiving the text-only instruction. Thus, to detect reduction in the error rate of 20% with a power of 80% and two-sided alpha of 0.05, the minimum sample size is approximately 300 patients, both for the sample as a whole and for subgroup analysis based on dosing literacy.

### Data collection

Mothers were invited to participate in a pre-measurement session and two measurement sessions-a baseline measurement session and an intervention session. Data were collected by interviewing the mother in paper forms comprising a questionnaire, which consisted of questions related to all three sessions.

### Pre-measurement

This section of the questionnaire assessed the sociodemographic characteristics and perception of the mothers about using measuring devices. The mothers were interviewed regarding the medication dosing devices they have used until now and to rate these devices for the ease of use and accuracy. The reason for their choice was explored in subsequent questions.

### Baseline measurement session

In the baseline measurement session all the participating mothers, irrespective of their literacy level, were read out instructions for measurement of a liquid medication in mL and drop format. The baseline measurement session was call oral only measurement session (OMS). The conditions under which this oral only measurement session was undertaken is mentioned later.

### Intervention

Pictogram to explain the liquid medication dosing so as to reduce dosing errors was the intervention. The intervention measurement session was called oral instruction plus pictogram measurement session (OPMS) [17]. In the OPMS the mothers were read out the instructions (as in the OMS) along with a pictographic illustration of the dose using a hand-drawn pictogram dosing diagram and were asked to remeasure the dose using the provided test–pictogram instruction (OPMS).

### Study tasks

Each mother participated in both OMS and OPMS and was asked to measure a specified dose of paracetamol of the same brand in drop and suspension using three dosing devices: a dropper for drops, and a measuring cup and calibrated spoon for suspension. The participants in each session measured: two doses with each device. Thus, each participating mother measured 12 times (Additional file 1: Table S1 and Additional file 2: Figure S1). The dose and device used to measure was randomly assigned for each participating mother.

### Study conditions

**Fig. 1:**
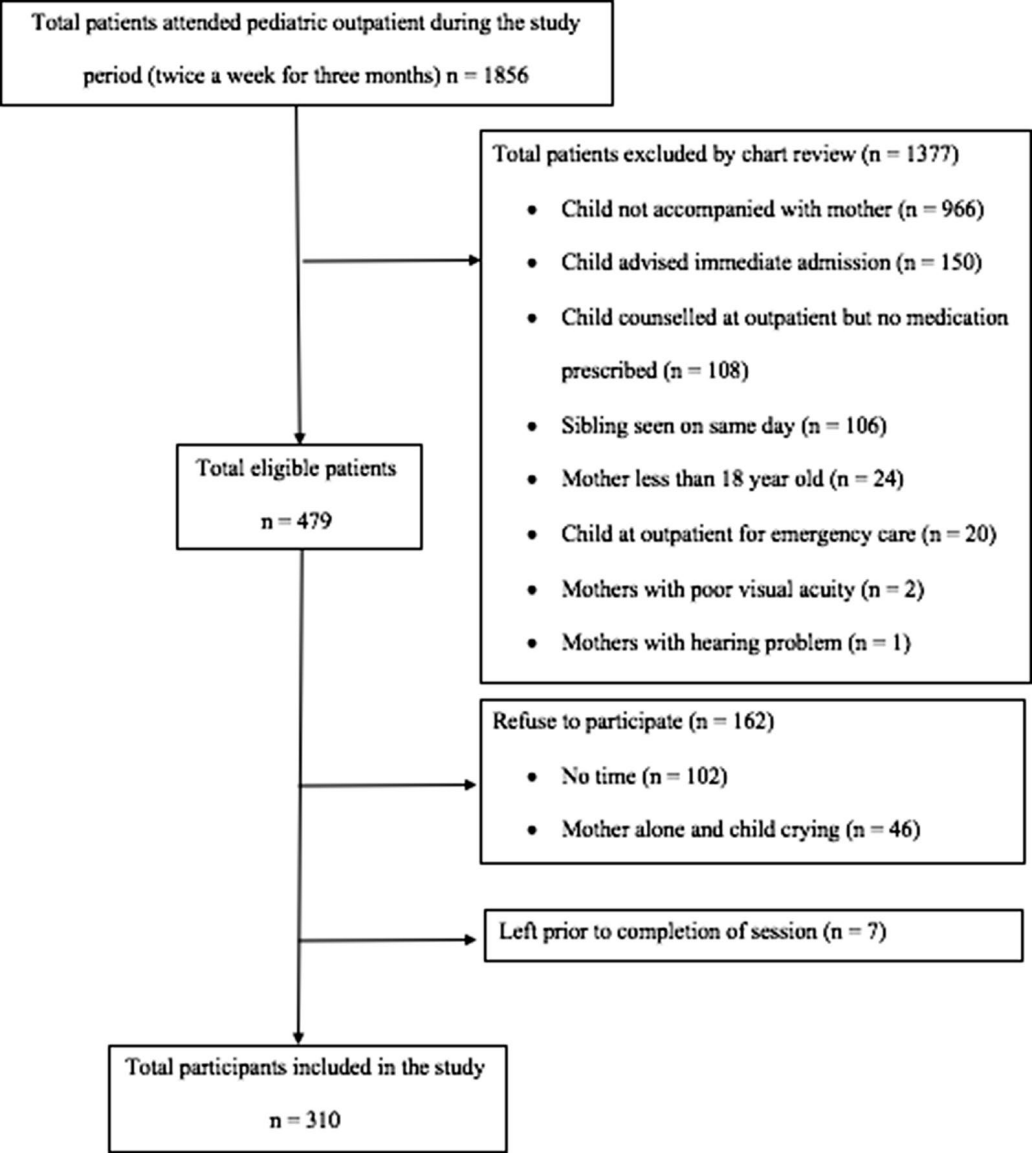
Flow chart of the recruitment process of the participants in the study

The instructions were read in the same sequence regardless of the medication dose during both OMS and OPMS. An example of pictograms used in the study is presented in Additional file 2: Figure S1. No communication took place between the mothers and researcher during mothers' task to remeasure the dose. No time limit was specified for completing the entire protocol. No incentive was provided to the caregiver for study participation (Fig. 1).

### Measurement of dosing error

The medication volume measured by the mother was recorded using a variable volume micropipette (Eppendorf India Private Limited, Chennai, India). The difference between the volume measured by the mother and the standard volume measured using the micropipette was used to calculate the measuring error.

### Fidelity of intervention

The principal investigator trained the research assistants to maintain the fidelity of the intervention. The intervention module consisted of a 3-h training session involving a discussion on all text messages and pictograms. The concepts were reinforced by allowing the research assistants to engage in role-playing. The session was repeated once every fortnight during the study period. A training manual was used to articulate the contents and delivery of the pictogram intervention. The mothers’ receipts of the intervention were assessed on the basis of reduction in underdosing or overdosing errors after the intervention.

### Outcome measure

Dosing error was the primary outcome variable; error magnitude was determined by volume measurement as defined above. A measurement deviation of > 20% was considered a dosing error. A large dosing error was defined as an error double of the threshold dosing error, viz. > 40% [20]. The measured dosing errors included underdosing or overdosing error.

### Statistical analysis

Data were entered using OpenEpi, Version 3 [21]. The data analysis was done using Stata (Version 12.0, Statacorp. Texas, USA). Descriptive statistics were calculated for each variable. Based on normalized data, baseline characteristics of the TPMS and TOMS were compared using paired t tests and one-way analysis of variance as appropriate. The significance level was set at *P* < 0.05.

Multivariate logistic regression was performed to investigate predictors of LMDE while controlling for relevant covariates. The dependent variable for multivariate logistic regression models was the device and volume identified with maximum error. Adjusted odds ratios (aORs) of the dose error for various covariates having a *P* value of < 0.1 were calculated using the backward multivariate logistic regression model. *P* < 0.05 was considered statistically significant.

### Ethical approval

The study was approved by the institutional ethical review board (IEC Ref. No- IEC/RDGMC/171).

## Results

The study included 310 mothers with a mean (± SD) age of 30.2 (± 4.18) years. The mean (± SD) size of the family was 4.94 (± 1.74) members, with the mean (± SD) number of children being 1.75 (± 0.79). The mean (± SD) age of the father was 34.38 (± 3.6) years. Table 1 presents the sociodemographic characteristics of the participating mothers. The most preferred dosing device for measuring liquid medications was the measuring cup (39%). The most common reason for selecting a dosing device was the ease of use (54%; n = 167). Table 2 presents other practices and perceptions of mothers regarding administering liquid medications to their children.

**Table 1:** Socio-demographic characteristics of the mothers and their families (n = 310) included in the study in Ujjain, India

Independent variables	n (%)^a^	Independent variables	n (%)^a^
*Locality*		*Mother occupation*	
Urban	120 (39)	Housewife	133 (43)
Rural	190 (61)	Self employed	30 (10)
*Home type*		Farm worker	126 (41)
Kuccha	65 (21)	Labor	7 (2)
Pukka	98 (32)	Office work	14 (4)
Kuccha-Pukka	147 (47)	*Father education*	
*Family type*		Uneducated-primary school	9 (3)
Nuclear	193 (62)	Middle school-higher secondary	160 (52)
Joint	117 (38)	Graduate-post graduate	141 (45)
*Number of children*		*Mother occupation*	
1	137 (44)	Unemployed	1 (1)
2	121 (39)	Self employed	146 (47)
> 2	52 (17)	Farm worker	132 (42)
*Overcrowding*		Labor	4 (2)
Yes	79 (25)	Office work	27 (8)
No	231 (75)		
*Mother education*			
Uneducated-primary school	106 (34)		
Middle school-higher secondary	150 (48)		
Graduate-post graduate	54 (17)		

^a^Column percentage

**Table 2:** Practices and perceptions of the mothers (n = 310) regarding administering liquid medication to their child

Variable	n (%)^a^
*Do you have past experience in giving liquid medication?*	
Yes	293 (95)
No	17 (5)
*Have you experienced difficulty in the last medication administration?*	
Yes	198 (64)
No	112 (36)
*What is the most common device preferred by you to measure liquid medication?*	
Calibrated spoon	9 (3)
Teaspoon	70 (22)
Syringe	8 (3)
Measuring cup	123 (39)
Dropper	28 (9)
Bottle cap	61 (20)
Direct sip from bottle	11 (4)
*What is the most important reason for choosing a measuring device?*	
The medicine can be measured accurately with the device	41 (13)
It is easy to find the device at home	56 (18)
Device is easy to use	167 (54)
Device reduces risk of spilling the medicine	46 (15)
*Did you adjust the dose according to physician instruction in the last medication administration?*	
Yes	146 (47)
No	164 (53)
*Did you have difficulty in understanding the dosing instruction given by the doctor?*	
Yes	270 (87)
No	40 (13)

^a^Column percentage

The percentage error in measuring the correct dose for 0.5, l, 2.5, and 5 mL by the dropper, cup, and calibrated spoon improved from 60%, 63%, 62%, 66%, 69%, and 68%, respectively, in the OMS to 12%, 9%, 8%, 7%, 12%, and 13%, respectively, in the OPMS. The corresponding ORs are listed in Table 3.

**Table 3:** Prevalence of dosing errors committed by mothers, binomial effect size display, odds ratios of reduction in dosing errors and Cohen’s d (effect size) of the pictogram intervention (TPMS)

Device	Doses (mL)	TOMS		TPMS		BSED (%)	OR** (95% CI)	*Effect size (d*_*cohen*_*)*
		Incorrect	Correct	Incorrect	Correct			
		n = 310 (%)^a^	n = 310 (%)^a^	n = 310 (%)^a^	n = 310 (%)^a^			
Dropper	0.5	185 (60)	125 (40)	38 (12)	272 (88)	48	10.55 (7.05–16.02)	1.86
	1	196 (63)	114 (37)	28 (9)	282 (91)	54	17.22 (11.07–27.44)	3.01
Measuring cup	2.5	192 (62)	118 (38)	26 (8)	284 (92)	54	17.68 (11.25–28.51)	2.89
	5	206 (66)	104 (34)	23 (7)	287 (93)	59	24.56 (15.3–40.65)	6.4
Calibrated spoon	2.5	215 (69)	95 (31)	38 (12)	272 (88)	57	16.11 (10.69–24.67)	3.83
	5	210 (68)	100 (32)	41 (13)	269 (87)	55	13.71 (9.19–20.75)	2.9

Text only measurement session (TOMS) and text plus pictogram measurement session (TPMS); BSED: Binomial effect size display (intervention success); *OR* odds ratios** (*P* < 0.001 for all OR displayed)

**Fig. 2:**
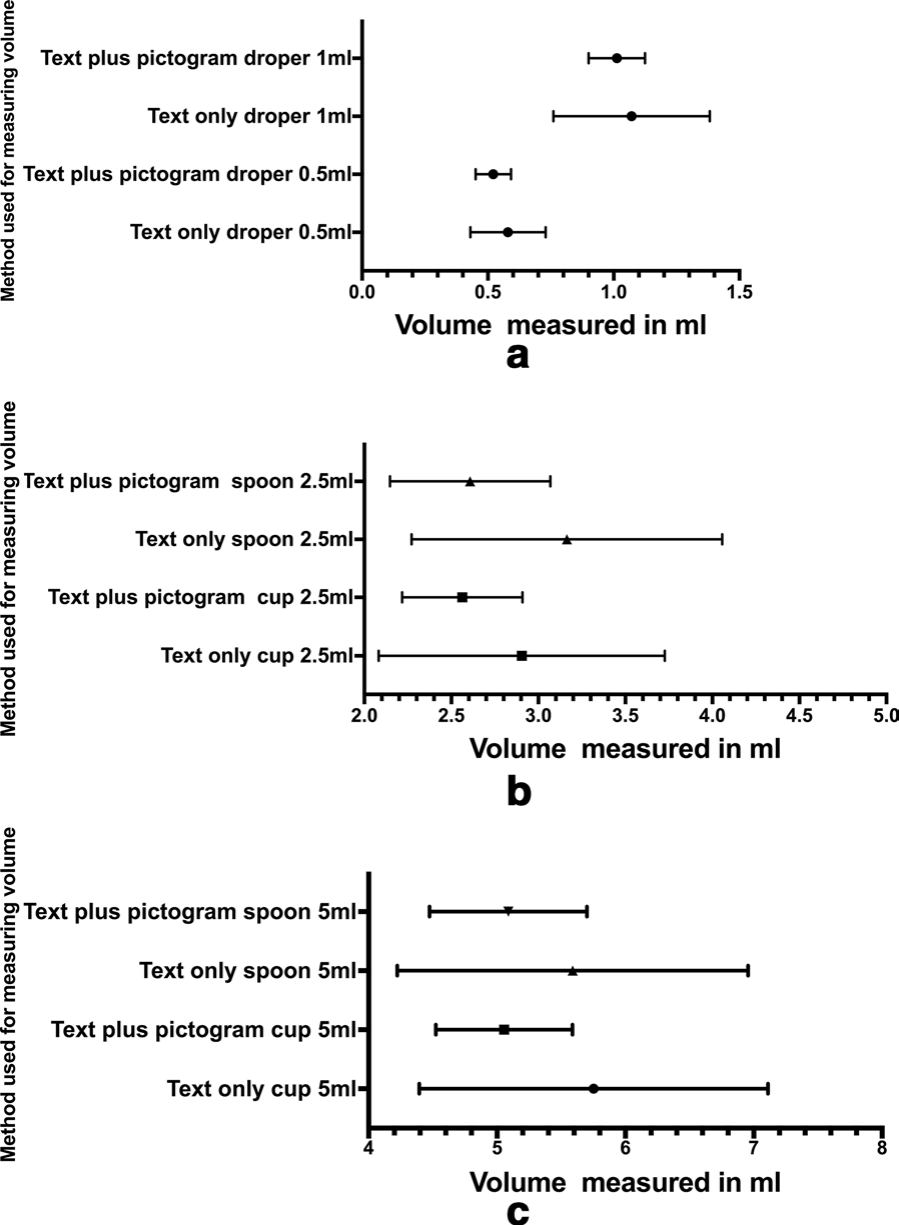
Whisker Box plots for actual volumes measured by the mother for different volumes and measuring devices

In the OMS, the prevalence of correct, overdose, and underdose for 0.5 and 1 mL using the dropper was 40%, 44%, and 15% and 37%, 39%, and 24%, respectively. The prevalence of correct, overdose, and underdose using the measuring cup was 38%, 46%, and 16% and 34%, 55%, and 12%, respectively (Table 4). The prevalence of correct, overdose, and underdose using the calibrated spoon is shown in Table 4. Figure 2 shows the Whisker-box plots of medication errors for all measuring devices and medication doses used in the study.

**Table 4:** Prevalence of overdose and under dose in mothers (n = 310) during measuring sessions

Dosing device	Dose (mL)	Session N = 310	Overdose n (%)	OR	Underdose n (%)	OR
Dropper	0.5	TOMS	137 (44)	7.12	48 (16)	7.93
		TPMS	31 (10)		7 (2)	
	1	TOMS	122 (39)	10.52	74 (24)	9.40
		TPMS	18 (6)		10 (3)	
Measuring cup	2.5	TOMS	142 (46)	13.71	50 (16)	4.95
		TPMS	18 (6)		8 (2)	
	5	TOMS	170 (55)	20.92	36 (12)	6.65
		TPMS	17 (5)		6 (2)	
Calibrated spoon	2.5	TOMS	179 (58)	13.24	36 (12)	4.39
		TPMS	29 (9)		9 (3)	
	5	TOMS	168 (54)	10.27	42 (14)	5.24
		TPMS	32 (10)		9 (3)	

*OR* odds ratio; all rate ratios were statistically significant; (*P* < 0.001 for all OR displayed)

The frequency of large dosing errors were maximum (91%, n = 163) when the calibrated spoon was used to measure 2.5 mL of paracetamol, therefore a logistic regression model was run to explore the independent factors associated with the device with maximum dosing error. Table 5 presents the multiple logistic regression model for predictors of errors while measuring a dose of 2.5 mL using the calibrated spoon, and the predictors included age of the mother, type of family—nuclear versus joint, education status of mother—uneducated and primary schooling versus middle school and secondary school versus graduate and postgraduate. The Receiver Operator Curve of the final model was 0.826, showing an excellent model fit. The Hosmer–Lemeshaw test showed that chi-square was 13.77 (*P* = 0.087). A high *P* showing good model calibration was observed. The proportion of variance explained by the model with three independent variables was 29.6 (R^2^).

**Table 5:** Multivariate analyses of dependent variable for dosing error for calibrated spoon measuring 2.5 mL in mothers (n = 310)

Variable	Adjusted OR	95% CI		*P* value
		Lower	Upper	
*Age in months*^a^	1.08	1.01	1.17	0.026
Family type				
Nuclear	Referent	Referent	Referent	Referent
Joint	2.83	1.44	5.56	0.002
Mothers education				
Uneducated-primary school	Referent	Referent	Referent	Referent
Middle school-higher secondary	0.34	0.16	0.73	0.006
Graduate-post graduate	0.03	0.01	0.08	< 0.001

^a^Adjusted for age

## Discussion

In this study, the prevalence of dosing errors ranged from 60 to 69% with the different measuring devices, namely dropper, cup, and calibrated spoon, and with different doses, namely 0.5, 1, 2.5, and 5 mL. In other studies, the prevalence of LMDE was more than 40% and ranged from 40 to 60% with different instruments and different doses [8, 22–26]. However, LMDE prevalence was higher in our study. A possible reason for the high error rate can be the low education status of mothers and their failure to understand the test-only instructions.

In the pre-measurement session, the mothers were asked to report a preferred liquid dose measuring device and the reason for their preference. The measuring cup was the most preferred device, followed by non-standardized tools such as kitchen teaspoons and bottle caps. The most common reason for selecting the dosing instrument was the ease of use, followed by easy availability. Similar findings were reported in a study where a self-report 5-item survey was used to assess participants’ perceptions and preferred dosing instruments, that is, syringes and dosing cups [27]. Most (87%) participants reported cups as the preferred tool, as it is easy to use [27].

Major dosing errors in our study were committed during the OMS when mothers measured small volumes. Maximum (69%) errors were observed with calibrated spoons. While measuring large volumes, equal number of dosing errors were committed with calibrated spoons and measuring cups (68% in both). A similar finding was reported in a study conducted by the American Association of Poison Control Centers [24]. In New York, a randomized controlled experiment reported that a total of 84% parents made ≥ 1 dosing error and that the errors were more observed with cups than with other dosing devices, especially for smaller doses [26]. Other studies have also reported that cups were the most common device used to measure small volumes [4, 25, 28]. A calibrated spoon was another dosing tool that was identified to be associated with the risk of dosing errors while measuring small doses of 2.5 mL. In the present study, majority of the dosing errors (69%; OR 16.11; *P* < 0.001) were committed with the calibrated spoon, and these results are similar to those of another study, which reported 50% of the participants committing error when using a calibrated spoon [29]. The possible reasons for the high error rate in our setting were that calibrated spoons are less frequently used by Indian mothers and very few Indian pediatric medications come with calibrated spoons. For a large dosing error while measuring small volumes (2.5 mL), calibrated spoons were superior to other dosing devices used in the study. Similarly, another study reported that calibrated devices are associated with higher risk of parents committing a large dosing error while measuring small volumes (< 5 mL) [27].

Poor maternal education results in poor health literacy. Health literacy and socioeconomic status of families were found to be strongly associated with dosing errors in studies [26, 30, 31]. The mothers in our study with a poor education status committed more errors than those with a better education status. Communication problems between patients and healthcare providers can significantly affect dosing errors [17, 32, 33]. A better communication between patients and providers can minimize dosing errors among mothers with a poor socioeconomic status and education status [17, 32, 33]. Improved health communication can prevent dosing error-induced adverse drug reactions by 72% [31].

In our study, pictogram interventions reduced dosing errors by 48–54% points and improved mothers’ understanding of dosing. Incorporating pictorial aids into verbal medication counseling or text-based instructions was more beneficial than using the single approach alone [34]. This finding was similar to those of other studies in which pictogram interventions significantly reduced dosing errors [34]. In our study, mothers who received text-only (versus text and pictogram) instructions made most dosing errors with 2.5 mL measurement when using a calibrated spoon [OR = 16.11 (10.69–24.67)]. A similar finding has been reported previously [34].

Potential strategies for reducing dosing errors include the use of a health literacy informed approach to improve healthcare provider communication concerning medication instructions with caregivers through advanced counseling strategies and standardized dosing instruments [4, 28]. Liquid medication delivery devices alone are insufficient in reducing medication errors as provider communication problems contribute to confusion about medication administration, particularly for complex instructions [10, 25].

## Limitations

The study is a cross-sectional analysis and therefore, we cannot draw conclusions on causality. The design also does not allow conclusion over mothers ability to learn, which would have required a follow-up design. There could be a potential learning effect of the oral instruction, on the pictogram intervention, however we believe that this effect was minimal as the order of measurement with various volumes and devices was random and the results of measuring errors were not discussed with the mothers. The effect of the pictogram intervention was measured immediately, and the study was not designed to measure long-term effects. By when the mothers would forget the pictogram and whether they would remember to use it in an emergency remain unclear. We did not include a control group as each mother acted as her own control. The study was performed as an experimental set-up in the outpatient settings of hospital and thus, might not reflect the home environment of the mothers. The dosing errors were assessed using hypothetical doses, which might not be the way the mothers dose at home. The mothers recruited in our study were predominantly from rural areas with low education, thus the results are not generalizable to other populations.

## Recommendations

Pictograms can help in parent information and empowerment needed to reduce medication errors, as they can be an effective mode of communicating correct doses to uneducated parents. However, reducing medication errors cannot be the sole responsibility of parents. The health professional including the prescribers and the pharmacists should be made aware of the medication errors by the parents and that simple tools like pictogram can significantly reduce them. Parents should be instructed to use calibrated devices and take care not to lose them. Pharmaceutical companies should provide extra calibrated devices with the liquid medications and additionally such devices should be available with pharmacies.

## Conclusions

Pictogram-based medication consultation can reduce the risk of LMDE by mothers. A pictogram can be an effective provider–parent communication tool for parents with a low education status. Health care workers should be sensitized regarding the problem of dosing errors and should be motivated to use strategies such as pictograms to decrease these errors.

## Supplementary Information

**Additional file 1:** **Additional Table 1.** Different measuring devices and the corresponding doses used in text only and text plus pictogram measuring session.

**Additional file 2:** **Additional Figure 1.** Measuring devices and their pictograms used in the study. Fig 1 a Picture of actual measuring devices used in the study; Fig 1 b and 1 c Pictograms of dropper for measuring 0.5 ml and 1 ml, respectively; Fig 1 d and 1 e Pictograms of measuring cups for 2.5 ml and 5 ml, respectively; Fig 1 f and 1 g Pictograms of calibrated spoons for measuring 2.5 ml and 5 ml, respectively.

## Data Availability

The datasets used and/or analyzed during the current study is available from the corresponding author on reasonable request.

## Abbreviations

LMDE: Liquid medication dosing errors
NCCMERP: American National Coordinating Council for Medication Error Reporting And Prevention
LMIC: Low-and-middle income countries
OPD: Outpatient department
TOMS: Text only measurement session
TPMS: Text plus pictogram measurement session
OR: Odds ratio
aOR: Adjusted odds ratio
