# Supplementary material for: Can use of pictograms reduce liquid medication administration errors by mothers? An interventional study
Source: BMC Psychol. 2021 Jun 25;9:99. doi: 10.1186/s40359-021-00584-9 (PMC8228905; doi:10.1186/s40359-021-00584-9)
Supplement: Supplementary file 1 — Additional file 1: Additional Table 1. Different measuring devices and the corresponding doses used in text only and text plus pictogram measuring session. [file 40359_2021_584_MOESM1_ESM.pdf]

Table 1-Different measuring devices and the corresponding doses used in text only and text plus pictogram measuring session

Text only measuring session (TOMS)		Text plus pictogram measuring session (TPMS)	
**Measuring device**	**Dose to be measured**	**Measuring device**	**Dose to be measured**
Dropper	0.5 ml	Dropper	0.5 ml
Dropper	1.0 ml	Dropper	1.0 ml
Measuring cup	2.5 ml	Measuring cup	2.5 ml
Measuring cup	5.0 ml	Measuring cup	5.0 ml
Table spoon	2.5 ml	Calibrated spoon	2.5 ml
Table spoon	5.0 ml	Calibrated spoon	5.0ml
