# Supplementary material for: Can use of pictograms reduce liquid medication administration errors by mothers? An interventional study
Source: BMC Psychol. 2021 Jun 25;9:99. doi: 10.1186/s40359-021-00584-9 (PMC8228905; doi:10.1186/s40359-021-00584-9)
Supplement: Supplementary file 2 — Additional file 2: Additional Figure 1. Measuring devices and their pictograms used in the study. Fig 1 a Picture of actual measuring devices used in the study; Fig 1 b and 1 c Pictograms of dropper for measuring 0.5 ml and 1 ml, respectively; Fig 1 d and 1 e Pictograms of measuring cups for 2.5 ml and 5 ml, respectively; Fig 1 f and 1 g Pictograms of calibrated spoons for measuring 2.5 ml and 5 ml, respectively. [file 40359_2021_584_MOESM2_ESM.pdf]

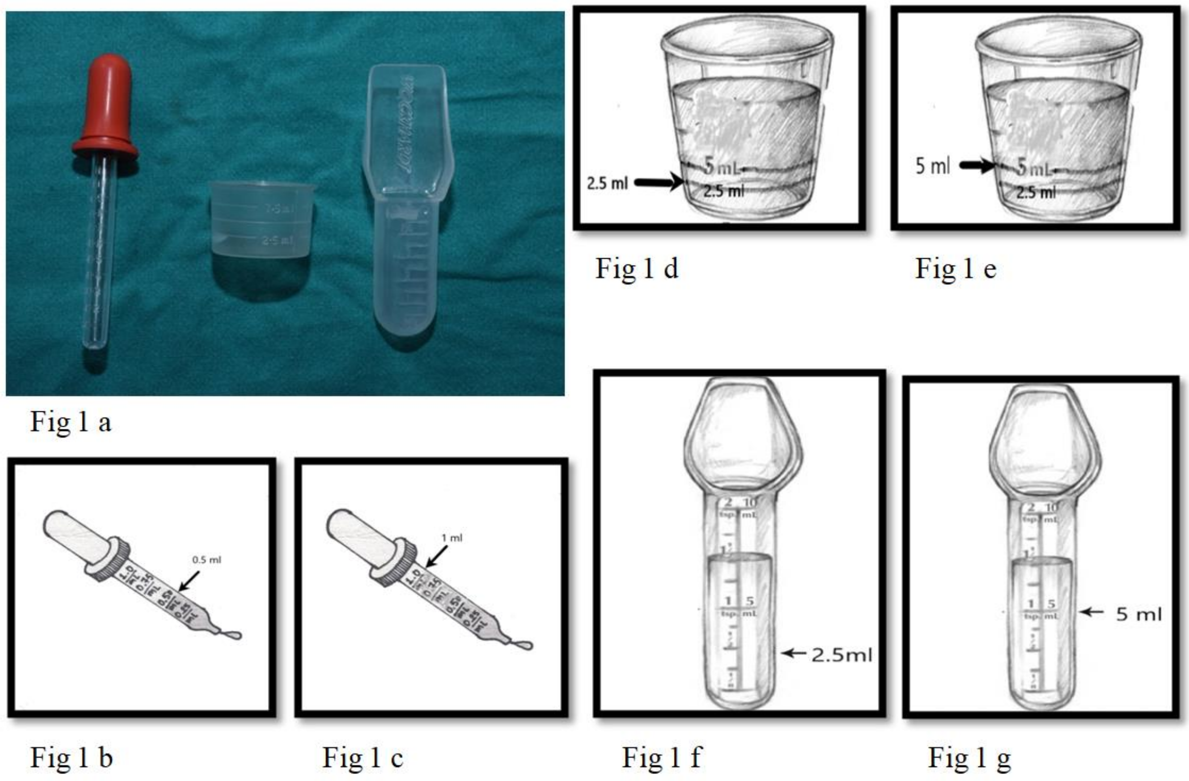

Additional Figure 1 Measuring devices and their pictograms used in the study. Fig 1 a Picture of actual measuring devices used in the study; Fig 1 b and 1 c Pictograms of dropper for measuring 0.5 ml and 1 ml, respectively; Fig 1 d and 1 e Pictograms of measuring cups for 2.5 ml and 5 ml, respectively; Fig 1 f and 1 g Pictograms of calibrated spoons for measuring 2.5 ml and 5 ml, respectively
